# Perception of problem based learning versus conventional teaching methods by clinical medical students in Nigeria

**DOI:** 10.11604/pamj.2019.33.311.19169

**Published:** 2019-08-20

**Authors:** Helen Chioma Okoye, Ijeoma Angela Meka, Angela Ogechukwu Ugwu, Isah Adagiri Yahaya, Ochuko Otokunefor, Olugbenga Olalekan Ojo, Emmanuel Onyebuchi Ugwu

**Affiliations:** 1 Department of Haematology and Immunology, College of Medicine, University of Nigeria, Ituku-Ozalla Campus, Enugu, Nigeria; 2 Department of Chemical Pathology, College of Medicine, University of Nigeria, Ituku-Ozalla Campus, Enugu, Nigeria; 3 Department of Haematology and Immunology, College of Medicine, University of Nigeria, Ituku-Ozalla Campus, Enugu, Nigeria; 4 Department of Chemical Pathology and Immunology, Bayero University/Aminu Kano Teaching Hospital, Kano, Nigeria; 5 Department of Chemical Pathology, College of Medicine, University of Port Harcourt, Rivers State, Nigeria; 6 Department of Surgery, Lagos University Teaching Hospital, Lagos, Nigeria; 7 Department of Obstetrics and Gynaecology, College of Medicine, University of Nigeria, Ituku-Ozalla Campus, Enugu, Nigeria

**Keywords:** Problem based learning, conventional teaching, medical students, pathology, Nigeria

## Abstract

**Introduction:**

Problem-based learning (PBL) method which was introduced about 50 years ago in Canada is beginning to gain acceptance over conventional teaching method (CTM) worldwide in medical education but still remains unpopular in Nigeria. This study aims to determine the perception of clinical medical students to the use of both learning methods in pathology courses.

**Methods:**

A cross-sectional quantitative survey was conducted in four Nigerian universities drawn from four regions of the country. Data were collected using pretested semi-structured self-administered questionnaires.

**Results:**

The study included 310 respondents, 182(58.7%) males and 128(41.3%) females. Of all the participants, 257(82.9%) had heard of PBL prior to the study and 260(83.9%) thought it suitable for teaching and learning Pathology. Majority of participants, 221(71.3%) preferred a combination of both PBL and CTM while 238(76.8%) thought PBL suitable for all medical students. Some identified factors capable of enhancing adaptation of PBL into medical curriculum include conducive quiet spaces for learning and availability of computers with internet facilities for students' use.

**Conclusion:**

Participants demonstrated high level of awareness of PBL and thought it suitable for all medical students. Availability of computers and up-to-date libraries with internet and audio-visual facilities could enhance adaptation of PBL into medical curriculum in Nigeria.

## Introduction

Pedagogy entails knowledge impaction on a learner through a well-defined learning process which lately is becoming complex due to evolution of ideas and societal changes [1]. This learning process involves a strategy of deliberately delivering a lesson in a professional manner in a bid to assist the learner to acquire skills and make perceptual changes [1,2]. The two major teaching methods are the problem-based learning (PBL) and the conventional teaching methods (CTM). The PBL is a student-centered learning method first introduced in Canada half a decade ago and involving critical thinking in order to solve problems aimed at empowering students to take more responsibility for their learning while increasing their motivation and sense of accomplishment [3,4]. On the other hand, CTM otherwise known as the traditional method of learning, is a much older learning method which in itself is teacher-centered where information which is not easily accessible is shared or delivered by the teacher in a quick manner, the students passively memorize facts often times and are not actively involved [5]. This teaching method appears to be of immense benefit to students who learn best by listening [5]. According to Awotua-Efebo, the essence of teaching is to assist students to gain, retain and utilize knowledge, develop the ability to criticize, synthesize and analyse issues while developing patterns and perspectives [1,6]. In order to achieve this purpose, one must consider the subject to be taught, the environment and the needs of individual students. The PBL is not yet popular as the official method of learning in Nigerian medical schools and to achieve better quality in medical schools, there has to be some tailoring of the curriculum to meet the exigencies of the students [7]. The curriculum in Nigerian medicals schools stems from basic (e.g. anatomy) to clinical (e.g. paediatrics) science courses through a mixture of both basic and clinical sciences (pathology). A few studies have been carried out to evaluate the teaching method in some clinical courses but rarely in pathology which is the only specialty with both basic and clinical aspects. This study aims to evaluate the perspectives of final year medical students towards methods of teaching or learning pathology.

## Methods

This was a descriptive cross-sectional study carried out among final year medical students in four Nigerian medical schools (each from one of the 4 regions of the country) in the following universities: University of Nigeria, UNN (East); University of Lagos, UNILAG (West); University of Port Harcourt, UNIPORT (South); and Bayero University, BUK, Kano (North); between March and October, 2018. The study population was recruited using total population sampling of all final year medical students of the four institutions. Informed consent was obtained from participants after the study aim was explained to them. Data were then collected using self-administered semi-structured questionnaires. The questionnaire was designed by the researchers, and pretested using ten students who eventually were not part of the study. The major questions in the questionnaire included, whether respondents had heard of PBL, if respondents think PBL is suitable for teaching and learning in Pathology, who PBL is best suited for, and factors that can enhance adaptation of PBL into the medical curriculum. Final year medical students who had not rotated through all arms of clinical medicine at the time of the study were excluded. Ethical clearance for the study was obtained from University of Nigeria College of Medicine Research Ethics Committee. Data obtained from the questionnaires were transferred to the statistical package for social sciences (SPSS) version 20.0 program, Chicago Illinois. Statistics was both descriptive and inferential. Continuous variables were presented as mean (SD), number and percentages while categorical variables were presented only as counts (number) and percentages. Chi square (Χ^2^) was used to compare categorical data. All tests were two-tailed with p < 0.05 taken as statistically significant.

## Results

Out of 418 questionnaires distributed to final year medical students in 4 different institutions, a total of 310 participated in the survey with a response rate of 74.2%. The participating institutions (and the number of respondents) were UNILAG (104, 33.5%), BUK (94, 30.3%), UNN (67, 21.6%) and UNIPORT (45, 14.5%). The participants consisted of 100(58.5%) males and 71(41.5%) females with a mean age of 22.4±3.6 yrs. These students were dichotomized into 3 different age groups: 20-25 years being the youngest followed by 26-30 years and then those >30 years of age representing the oldest age group. Majority of respondents were within the 20-25 age group (222, 71.6%) followed by the 26-30 age group (80, 25.8%). Only 8(2.6%) of the students were more than 30 years at the time of the study. Two hundred and fifty seven (82.9%) participants had heard of PBL prior to the study. After a brief overview of what PBL is all about, 260(83.9%) of the respondents thought it was suitable for teaching and learning pathology. Comparison of the different learning methods revealed that majority (221, 71.3%) preferred a combination of both PBL and CTM, 63(20.3%) preferred using PBL alone, while 26(8.4%) students found CTM preferable. Table 1 shows the participants' preferences according to the individual pathology courses.

**Table 1:** methods preferred by respondents for pathology courses (N=310)

Pathology Courses	PBL		CTM		COMBINATION	
	N	%	N	%	N	%
Chemical pathology	73	23.5	46	14.8	191	61.7
Haematology	69	22.3	40	12.9	201	64.8
Microbiology	51	16.5	65	21.0	194	62.5
Morbid Anatomy	55	17.7	42	13.5	213	68.8

Concerning the opinion of the respondents on whom PBL was best suited for, it was found that 238 (76.8%) thought it suitable for all medical students. Majority (298, 96.1%) affirmed that PBL was not just for extremely gifted students or only for students who voluntarily opted for PBL (266, 85.8%). Conducive quiet spaces for learning, availability of computers with internet facilities for students' use, well equipped and user-friendly laboratories and up-to-date libraries with audio-visual facilities were the factors identified by the students which could enhance adaptation of PBL into medical curriculum. Development of stronger problem solving skills (66.1%), higher retention ability and development of better attitude towards learning (53.9%) were the listed benefits of PBL while over half (165, 53%) of the respondents believed that development of team spirit was not a benefit. Students not being sure of the extent of what to learn (59.7%) and demands for longer teaching time (49.7%) were the major disadvantages perceived by respondents to be associated with PBL, whereas over four-fifth (82.6%) of them were of the opinion that higher cost of learning was not a problem. As shown in Table 2, the proportion of respondents who preferred PBL as a method for teaching was higher among those in the age group 20-25 years (54.8%), while only one respondent above age of 30 years preferred PBL. In the age group 20-25 years, 53.8% preferred CTM as a method of teaching, while 3.9% of those above age 30 years preferred CTM. The differences observed among age groups and institutions were statistically significant (P<0.001) while that observed among gender were not statistically significant (P=0.386). Details are as shown in Table 2.

**Table 2:** relationship of age, sex, and institutions to teaching methods preferred by the respondents (N=310)

Variables	Methods of teaching preferred						X^2^	P
	PBL		CTM		Combination			
	N	%	N	%	N	%		
**Age (Years)**								
20-25	34	54.8	14	53.8	174	78.4	19.062	<.001
26-30	27	43.5	11	42.3	42	18.9		
>30	1	1.7	1	3.9	6	2.7		
Total	62	100	26	100	222	100		
**Sex**								
Male	39	61.9	12	46.2	131	59.3	1.904	0.386
Female	24	38.1	14	53.8	90	40.7		
Total	63	100	26	100	221	100		
**Institution**								
UNN	10	15.9	6	23.1	51	23.1	44.089	<0.001
UNILAG	19	30.2	9	34.6	76	34.4		
BUK	11	17.4	3	11.5	80	36.2		
UNIPORT	23	36.5	8	30.8	14	6.3		
Total	63	100	26	100	221	100		

## Discussion

Four Nigerian Universities, each from one of the major regions of the country: BUK from the North, UNILAG from the West, UNN from the East and UNIPORT from the South were involved in the present study. This makes the study population a good representation of the nation. There was a slight male preponderance in the study. This is in keeping with previous researches revealing that there were more male enrollees than females in Nigerian universities [8,9] and more specifically, fewer females were enrolled for science courses than their male counterparts [9]. An explanation to this finding is not clear but may be as a result of the different perceptions of these courses by the different genders and the extent of their preparedness in lower levels of education. The mean age was found to be slightly lower than an earlier report by Bakare [10] which gave a mean age of 25 years. While universities in Sokoto and Kano were used in that study, only Kano was used in the present study and this may account for the higher mean age. Over four-fifth of the respondents had heard of PBL as a learning method. This high awareness rate in PBL may stem from the fact that clinical medical training uses a lot of case studies. Students are also made to undergo some levels of medical clerkship under the supervision of trainers. This is quite similar to what is entailed in PBL. Majority of the students thought it suitable for learning/teaching Pathology in medical schools. Pathology is a diverse discipline encompassing several subspecialties and may be defined as the study of the mechanisms underlying the disease processes [11]. While students are known to do better in basic sciences if they were taught using the CTM, those who learn using the PBL method do better in clinical sciences [12-15]. It is not surprising that respondents preferred a combination of both PBL and CTM in learning Pathology because Pathology is a discipline which encompasses both basic and clinical science aspects. Since CTM involves passing information from the teacher to the learner in a faster manner, [1] one would expect it to be the preferred learning method in Pathology given the large curriculum expected to be taught in a short time, but for education to be successful, the learner is expected to gain, retain and utilize the knowledge [2,5,6]. PBL concept is centered around students being able to learn independently outside of class time thereby freeing up time for critical analysis and group discussion and it is associated with higher retention rates [1,2].

Even though Han pointed out in a study that PBL approach is particularly appropriate for well-gifted students because it is effective in inducing their motivations, [16] it should not be restricted to such students because it is a teaching method that leads to development of deep critical thinking in the students while stimulating the development of problem solving skills and knowledge retention [1,2]. This is reflected in the opinion of majority of the study participants who indicated that PBL is suited for all medical students and not just for extremely gifted ones. Adaptation of PBL may be challenging giving the nature of the learning method; students are divided into smaller groups, given a problem to solve and each expected to assume an active role [1]. They take charge of their learning by determining the pace and extent, hence they would require a conducive space as well as access to information source to be effective [17]. As expected, the study participants listed conducive quiet spaces for learning, availability of computers with internet facilities, well equipped and user-friendly laboratories and up-to-date libraries with audio-visual facilities as factors that could enhance adaptation of PBL into medical curriculum. Through effective implementation of PBL, students are expected to develop a stronger problem solving skills, higher retention ability and better attitude towards learning, these were rightly listed by the participants as benefits of PBL. Inasmuch as PBL is student centered, the tutor plays a pivotal role in facilitating the learning process by directing and guiding the students [18]. Despite the numerous benefits, PBL is not without disadvantages [17]. When not properly applied or guided, students may face problems of not being sure of the extent of what to learn with consequent demands for longer learning time. These problems as noted by the respondents are in keeping with those documented by Woods and Vemon [17,19]. However, contrary to one of Wood's statements, majority of the respondents opined that higher cost of learning was not a problem.

It has been noted that while male students tend to preside over and direct discussions more than females who in turn are inclined to listening more, the females appear more sensitive to interpersonal dynamics [20]. Since PBL involves a lot of discussion and interaction, it is natural that males may tend to prefer PBL over CTM. Even though there was no significant association, the present study supports the supposition that gender may impact on students' preference for either PBL or CTM. It is noteworthy however that Sahu *et al.* found no gender difference in students' attitude to learning [21]. Despite the report from a previous study that age had no impact on PBL, [21] it is reasonable to argue that older individuals may benefit more from PBL when compared to the younger ones because they are able to apply their life experiences in discussions. The present study showed that respondents in the younger age group were more likely to embrace PBL than the CTM. This may not be unconnected to the fact that majority of the respondents are in the youngest age group, and this could have influenced the proportion that preferred PBL.

**Strengths and weaknesses of study:** being a cross-sectional study, causality of findings could not be determined. Participants' source of information on PBL was not determined in this study, and could be addressed by further studies on this subject. Despite this, the present study is very relevant as it contributes and provides much needed data concerning PBL in Nigerian medical schools. Another major strength of the present study is the multi-center design, drawing representatives from the four major geographical regions of Nigeria.

## Conclusion

Most respondents were aware of PBL and thought it suitable for learning Pathology because it could lead to developing stronger problem solving skills, higher retention ability and better attitude towards learning. Availability of computers and up-to-date libraries with internet and audio-visual facilities could enhance adaptation of PBL adaptation into medical curriculum. Problem of not knowing the extent of learning and demand for longer learning time may be some disadvantages.

### What is known about this topic

- Problem-based learning is a newer teaching method compared to conventional teaching method;
- Problem-based learning is gradually gaining acceptance over conventional teaching method worldwide in medical education;
- Problem-based learning is still unpopular in Nigeria.

### What this study adds

- Majority of the participants indicated that problem-based learning was suitable for teaching and learning Pathology and equally suitable for all medical students, not just for extremely gifted students;
- Majority of participants however preferred a combination of both problem-based learning and conventional teaching method in teaching and learning;
- More respondents in the age group 20-25 years preferred problem-based learning over conventional teaching method as a teaching and learning method.

## Competing interests

The authors declare no competing interests.
